# Assessing clustering of metabolic syndrome components available at primary care for Bantu Africans using factor analysis in the general population

**DOI:** 10.1186/1756-0500-6-228

**Published:** 2013-06-12

**Authors:** John Nasila Sungwacha, Joanne Tyler, Benjamin Longo-Mbenza, Jean Bosco Kasiam Lasi On'Kin, Thierry Gombet, Rajiv T Erasmus

**Affiliations:** 1 Department of Statistics, Walter Sisulu University, Mthatha, South Africa; 2 Department of Statistics, University of Forte Hare, Eastern cape, South Africa; 3 Faculty of Health Sciences, Walter Sisulu University, Private Bag X1, Mthatha, Eastern Cape, 5117, South Africa; 4 Department of Internal Medicine, University of Kinshasa, Kinshasa, DR Congo; 5 Division of Cardiology and Intensive Care, University of Maiden Ngouabi, Kinshasa, Congo; 6 Emergency Department, University Hospital Center, Brazzaville, Congo; 7 Division of Chemical Pathology, Stellenbosch University, Cape Town, Stellenbosch, South Africa

**Keywords:** Factor analysis, Metabolic syndrome, Black Africans, Type 2 diabetes

## Abstract

**Background:**

To provide a step-by-step description of the application of factor analysis and interpretation of the results based on anthropometric parameters(body mass index or BMI and waist circumferenceor WC), blood pressure(BP), lipid-lipoprotein(triglycerides and HDL-C) and glucose among Bantu Africans with different numbers and cutoffs of components of metabolic syndrome(MS).

**Methods:**

This study was a cross-sectional, comparative, and correlational survey conducted between January and April 2005, in Kinshasa Hinterland, DRC. The clustering of cardiovascular risk factors was defined in all, MS group according to IDF(WC, BP, triglycerides, HDL-C, glucose), absence and presence of cardiometabolic risk(CDM) group(BMI,WC, BP, fasting glucose, and post-load glucose).

**Results:**

Out of 977 participants, 17.4%( n = 170), 11%( n = 107), and 7.7%(n = 75) had type 2 diabetes mellitus(T2DM), MS, and CDM, respectively. Gender did not influence on all variables. Except BMI, levels of the rest variables were significantly higher in presence of T2DM than non-diabetics. There was a negative correlation between glucose types and BP in absence of CDM. In factor analysis for all, BP(factor 1) and triglycerides-HDL(factor 2) explained 55.4% of the total variance. In factor analysis for MS group, triglycerides-HDL-C(factor 1), BP(factor 2), and abdominal obesity-dysglycemia(factor 3) explained 75.1% of the total variance. In absence of CDM, glucose (factor 1) and obesity(factor 2) explained 48.1% of the total variance. In presence of CDM, 3 factors (factor 1 = glucose, factor 2 = BP, and factor 3 = obesity) explained 73.4% of the total variance.

**Conclusion:**

The MS pathogenesis may be more glucose-centered than abdominal obesity-centered in not considering lipid-lipoprotein , while BP and triglycerides-HDL-C could be the most strong predictors of MS in the general population. It should be specifically defined by ethnic cut-offs of waist circumference among Bantu Africans.

## Background

Metabolic syndrome (MS) is defined by a cluster of cardiovascular risk factors such as obesity (abdominal obesity in particular), diabetes mellitus (DM), high blood pressure (BP)/hypertension, dyslipidemia, insulin resistance, and hypercoagulability [1-5]. Furthermore, MS is nowadays a major public health problem worldwide [6]. Before the advent of the consensus of the definition of the MS using International Diabetes Federation(IDF) [6], Experts from WHO and EGIR required the measurement of serum insulin levels, diabetes mellitus(DM) or glucose intolerance, hypertension, triglycerides, HDL-Cholesterol(C), body mass index(BMI), and waist circumference(WC) with different cutoff points [7,8]. However, MS characterizes by international cutoff points [6-9], are limited to detect efficiently bantu Africans at higher cardiometabolic(CDM) risk(hypertension, DM, atherosclerosis) in comparisons with ethnic specific definition of MS [10]. Africans have low or normal lipid profile [11,12] and a high level of HDL-C [13]. Longo-Mbenza et al. include low birth weight, coronary heart disease, malnutrition, elevated fibrinogen, total cholesterol, and urea nitrogen [11-13].

In sub-Saharan Africa, MS, obesity, dyslipidemia, DM, hypertension and DM are emerging with cardiovascular complications [14-21] because of urbanization, migration, epidemiologic transition, demographic transition, and nutrition transition [22-24]. Identifying patterns of Bantu Africans at the primary care level can explain, at least in part, the differences observed in the prevalence or incidence of MS and cardiovascular diseases between different populations [25-30].

Several statistical methods can be used to identify patterns of clustering in cardiovascular diseases such as DM and hypertension. One such important and useful technique is factor analysis – a multivariate technique [31-36]. Indeed, Factor analysis is a statistical method used to describe variability among observed variables in terms of a potentially lower number of unobserved variables called factors. Factor analysis searches for such joint variations in response to unobserved latent variables. The observed variables are modelled as linear combinations of the potential factors, plus "error" terms. The information gained about the interdependencies between observed variables can be used later to reduce the set of variables in a dataset. Furthermore, at our knowledge, there is no information on the physiogenic process including the mechanisms with which the major components of the MS relate to each other which could be one of the features to make preventive startegies and control of emerging cardiovascular diseases in Africa [17-20]. For that reason, the objective of this study was to provide a step-by-step description of the application of factor analysis and interpretations of the results based on the clustering of anthropometric parameters, blood pressure, triglycerides, HDL-C, and plasma glucose in all, presence of MS defined by IDF, absence and presence of CDM(exclusion of triglycerides and HDL-C).

## Methods

This study was a cross-sectional survey conducted between January, and April 2005, in Kinshasa Hinterland with details previously published [13]. This study was carried out in compliance with the Helsinki Declaration(59^th^ WMA General Assembly, Seoul, South Korea, October 2008. http://www.wma.net/en/30publications/10policies/b3/index.html). This research was approved by the Ethics Committee of Lomo Medical Clinic(Ref-00038-03-07) at Kinshasa Limete. Fully informed and written consent was obtained from all adult participants.

The survey was specifically and extensively designed using a statistical multistage and stratified random model at each level to recruit a study sample with similar and representative characteristics of Kinshasa Hinterland demographic and socioeconomic structure and results comparable with global data on DM.

Each region contributed with a number of cluster (EDs) calculated by population number: 185, 112 inhabitants for the upper urban area of Gombe, 161,410 inhabitants of the semi-rural Kisero area, 153,265 inhabitants for the urban Lukemi area and 146,034 inhabitants for the deepest rural Feshi area. The sample size was calculated as Z^2^xPxQx the expected prevalence of DM in each area, Q = 1-P, d is the in the absolute accuracy of 2% ad f = 8.5 to correct the design effect.

The details of collection of weight, height, waist circumference (WC), systolic blood pressure (SBP), diastolic blood pressure (DBP), plasma fasting glucose and plasma post load glucose have been described elsewhere [30-32].

### Definitions

Body mass index (BMI) was obtained in dividing weigh (kg) by height (m)^2^. In our setting with limited resources and lack of routinely measured insulin resistance (gold standard), we applied the criteria of MS diagnosis proposed by the International Diabetes Federation (IDF) as follows: raised systolic blood pressure (SBP > 130 mmHg) and diastolic blood pressure (DBP > 85 mmHg), elevated triglycerides (TG > 1.7 mmol/L), low high-density lipoprotein cholesterol (HDL < 1.04 mmol/L in men and <1.29 mmol/L in women) levels, abdominal obesity defined by increased waist circumference (WC > 94 cm in men and >80 cm in women), and fasting plasma glucose (FPG > 5.6 mmol/L)(6).

CDM was defined by the constellation of 3 components of WHO – defined MS such as diabetes, hypertension, and BMI > =30 kg/m2. However, absence of CDM was defined in participants without pre-hypertension, abdominal obesity, BMI > =25 kg/m2, and CDM. The definition of diabetes was based on clinical arguments and the latest WHO/IDF criteria among persons with the fasting venous plasma glucose level > =126 mg/dL or Post-load venous blood plasma level > =200 mg/dL [7]. This was an undiagnosed T2DM so that information about HbA1c, duration of diabetes, and medications was not available and compulsory.

### Statistical analysis

Data were presented as *mean* ± *SD*. Factor analysis originated in *psychometrics*, and is used in behavioral sciences, *social sciences, marketing, product management, operations research*, and other applied sciences that deal with large quantities of data.

### Factor analysis is based on the following statistical model and definitions

Suppose we have a set of *p* observable random variables, x_1_,…,x_*p*_ with means. *μ*_*1*_*,…,μ*_*p*_.

Suppose for some unknown constants *l*_*ij*_ and *k* unobserved random variables *F*_*j*_, where i ϵ 1,…, *p* and j ϵ 1,…, where *k* < *p*, we have

$$x_{i}-{µ}_{i}=l_{i1}F_{1}+\cdot \cdot \cdot +l_{\mathrm{ik}}F_{k}+\epsilon_{i}.$$

Here, the *ϵ*_i_’s are independently distributed error terms with zero mean and finite variance, which may not be the same for all *i*. Let **Var(*ϵ***_***i***_**)*ψ***_***i***_, so that we have

$$\mathrm{Cov}\left(\epsilon \right)=\mathrm{Diag}\left(\psi_{1},\ldots ,\psi \right)=\Psi \;\mathrm{and}\;E\left(\epsilon \right)=0.$$

In matrix terms, we have

x−µ=LF+ϵ.

If we have *n* observations, then we will have the dimensions x_*p*×*n*_, *L*_*p*×*k*_, and *F*_*k*×*n*_. Each column of *x* and *F* denote values for one particular observation, and matrix *L* does not vary across observations.

Also we will impose the following assumptions on *F*.

1. *F* and *ϵ* are independent.

2. E(*F*)=0

3. Cov(*F*)=*I*

Any solution of the above set of equations following the constraints for *F* is defined as the *factors*, and *L* as the *loading matrix*.

Suppose Cov(*x* − μ) = Σ. Then note that from the conditions just imposed on *F*, we have

$$\text{Cov}\left(x-µ\right)=\text{Cov}\left(\mathrm{LF}+\epsilon \right),$$

or

$$\sum =L\text{Cov}\left(F\right)L^{T}+\text{Cov}\left(\epsilon \right),$$

or

$$\sum =LL^{T}+\Psi .$$

Note that for any Orthogonal Matrix *Q* if we set *L*=*LQ* and *F*=*Q*^*T*^*F*, the criteria for being factors and factor loadings still hold. Hence a set of factors and factor loadings is identical only up to orthogonal transformations.

*Common factor analysis*, also called principal factor analysis (PFA) or principal axis factoring (PAF), seeks the least number of factors which can account for the common variance (correlation) of a set of variables.

Analogous to Pearson's r, the squared factor loading is the percent of variance in that indicator variable explained by the factor. To get the percent of variance in all the variables accounted for by each factor, the sum of the squared factor loadings for that factor (column) was added and divided by the number of variables. This is the same as dividing the factor's Eigenvalue by the number of variables.

The Eigenvalue for a given factor measured the variance in all the variables which is accounted for by that factor. Eigenvalues measure the amount of variation in the total sample accounted for by each factor.

*Extraction sums of squared loadings were* performed. Factor scores were the scores of each case (row) on each factor (column). To compute the factor score for a given case for a given factor, the case's standardized score was taken on each variable, multiplied by the corresponding factor loading of the variable for the given factor; and these products were summed.

For determining the number of factors, the Kaiser criterion was used. The Kaiser rule is to drop all components with Eigenvalues under 1.0.

The Cattell scree test plotted the components as the X axis and the corresponding Eigenvalues as the Y-axis. As one moves to the right, toward later components, the Eigenvalues drop. When the drop ceases and the curve makes an elbow toward less steep decline, Cattell's scree test says to drop all further components after the one starting the elbow.

Varimax Rotation served to make the output more understandable and facilitated the interpretation of factors. This is an orthogonal rotation of the factor axes to maximize the variance of the squared loadings of a factor (column) on all the variables (rows) in a factor matrix, which has the effect of differentiating the original variables by extracted factor. This procedure yields results which make it as easy as possible to identify each variable with a single factor. To avoid theoretical supposed grounds, we used oblique Promax rotation as additional alternative to varimax rotation for suited clustering characteristics.

A P-value < 0.05 was considered as statistically significant. All analyses were performed using the Statistical Package for Social Sciences (SPSS) for windows version 18.0 (SPSS Inc) Chicago, Il, USA.

## Results

Out of the original population (n = 977 with 458 males and 519 females), 170(17.4%), 107(11%), and 75(7.7%) were diagnosed for new T2DM, MS, and CDM, respectively.

Table 1 describes the mean levels of general characteristics according to T2DM status. Except similar(P > 0.05) values of BMI in presence and absence of T2DM, levels of age, WC, SBP, DBP, and triglycerides were significantly(P < 0.05) higher in T2DM participants than no diabetic participants. However, HDL-C values were significantly(P < 0.05) lower in T2DM presence than diabetes absence. The mean levels of age, BMI, WC, SBP, DBP, triglycerides, HDL-C, FPG,and post-load glucose in men were similar(P > 0.05) with those from women(results not shown).

**Table 1:** Association of age and metabolic syndrome components with incident type 2 diabetes mellitus in the study population

**Variables of interest**	**Participants with incident types 2 DM**	**Absence of diabetic participants**	**P Values**
Age (Years)	53 ± 13	36 ± 12	< 0.0001
BMI (Kg/m^2^)	24.4 ± 5.6	23.3 ± 5.4	0.881
WC (cm)	86 ± 14.7	78.7 ± 14.3	< 0.0001
SBP (mm Hg)	131.1 ± 31.1	117 ± 17	< 0.0001
DBP (mm Hg)	80 ± 16.7	70 ± 11.7	< 0.0001
FPG (mmd/L)	7.5 ± 2	5.1 ± 1	< 0.0001
Triglycerides (mmd/L)	3.8 ± 0.8	2.7 ± 0.9	< 0.0001
HDL – C (mmd/L)	1 ± 0.3	2 ± 0.6	< 0.0001

Retrieved from “http://en.wikipedia.org/wiki/Factor_analusis.”

In the general population, factor analysis generated 2 factors which were explaining 55.4% of total variance: factor 1(Blood pressure with variance = 29.6%; DBP = 0.881 and SBP = 0.872), and factor 2(Dylipidemia with variance = 25.8%; HDL-C = -0.886 and triglycerides = 0.872).

In MS participants, factor analysis generated 3 factors with total variance of 75.1%: factor 1(Dyslipidemia with variance = 29.5%; triglycerides = 0.911 and HDL-C = -0.874), factor 2(Blood Pressure with variance = 27.9%; DBP = 0.869 and SBP = 0.837), and factor 3(Abdominal obesity + Dysglycemia with variance = 18.1%; WC = 0.836 and fasting plasma glucose = 0.609).

### Absence of CDM(n = 572)

Table 2 describes the mean values of variables analyzed in participants without CDM. The correlation matrix in absence of CDM is presented in Tables 3 and 4. Post-load plasma glucose was significantly and positively correlated to BMI and WC, but significantly and negatively correlated to both SBP and DBP. SBP was significantly and positively correlated to BMI but significantly but negatively correlated to FPG. DBP was significantly and negatively correlated to FPG.

**Table 2:** C**haracteristics in absence of cardiometabolic risk**

**Variables**	**Mean±SD**
BMI (Kg/m^2^)	24.6 ± 8.80
Waist Circumference (CM)	79.8 ± 13.6
SBP (mmHg)	113.4 ± 12.4
DBP (mmHg)	66.6 ± 07.6
FPG (mg/dL)	82.0 ± 14.0
Post-Load PG (mg/dL)	123.21 ± 18.0

**Table 3:** Correlation matrix in absence of cardiometabolic risk

	**WC**	**FPG**	**Post-load PG**
BMI	-	-	0.132
			P = 0.016
SBP	-	-	−0.114
			P = 0.031
DBP	-	-	−0.125
			P = 0.020
WC		0.035	0.146
		P = 0.286	P = 0.008

**Table 4:** Correlation matrix in absence of cardiometabolic risk

	**BMI**	**SBP**	**DBP**
BMI	-	0.104	0.052
		P = 0.046	P = 0.197
SBP	-	-	0.128
			P = 0.019
DBP	-	-	-
WC	0.200	0.063	−0.021
	P < 0.0001	P = 0.286	P = 0.364
FPG	−0.086	−0.155	−0.118
	P = 0.080	P = 0.006	P = 0.027

**Figure 1:**
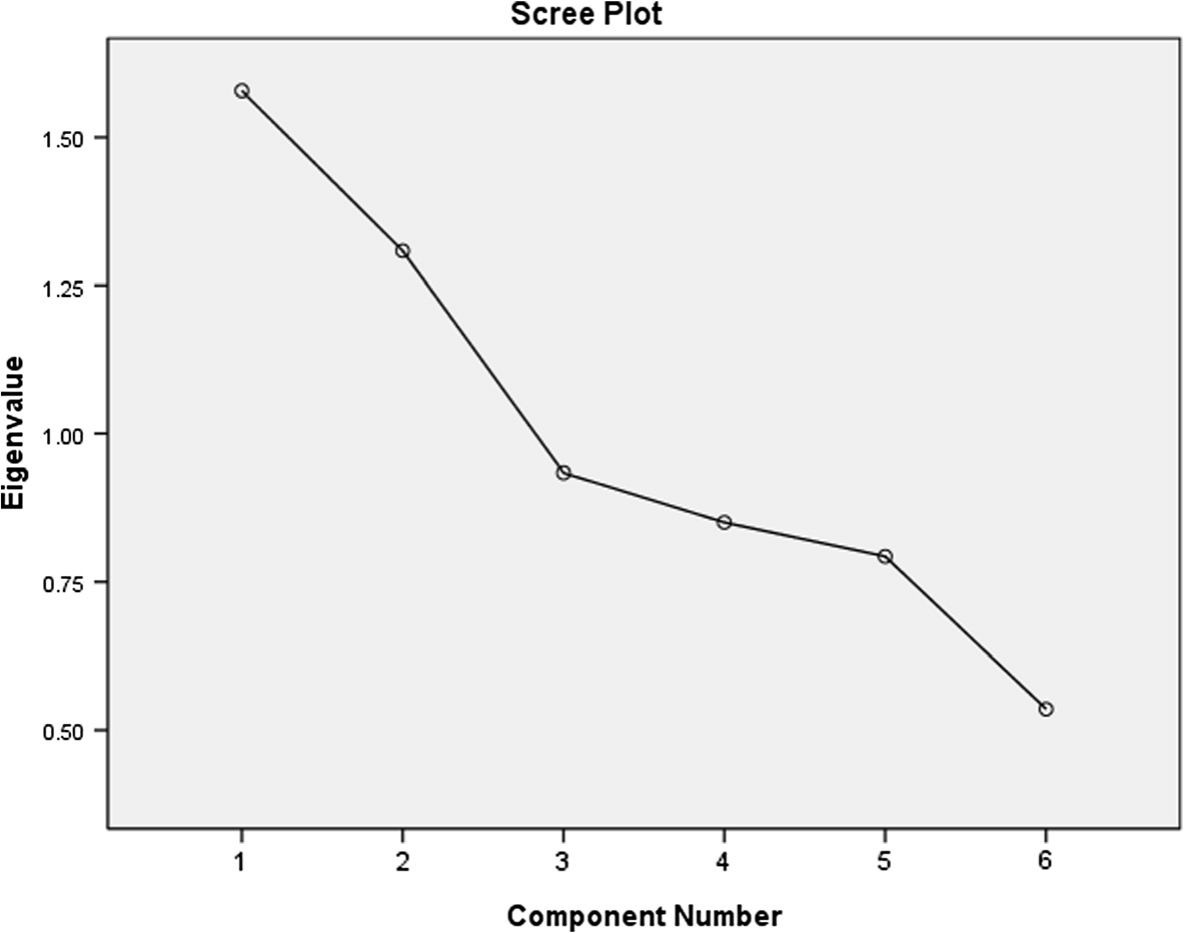
Eigen values among participants without cardio-metabolic risk.

**Figure 2:**
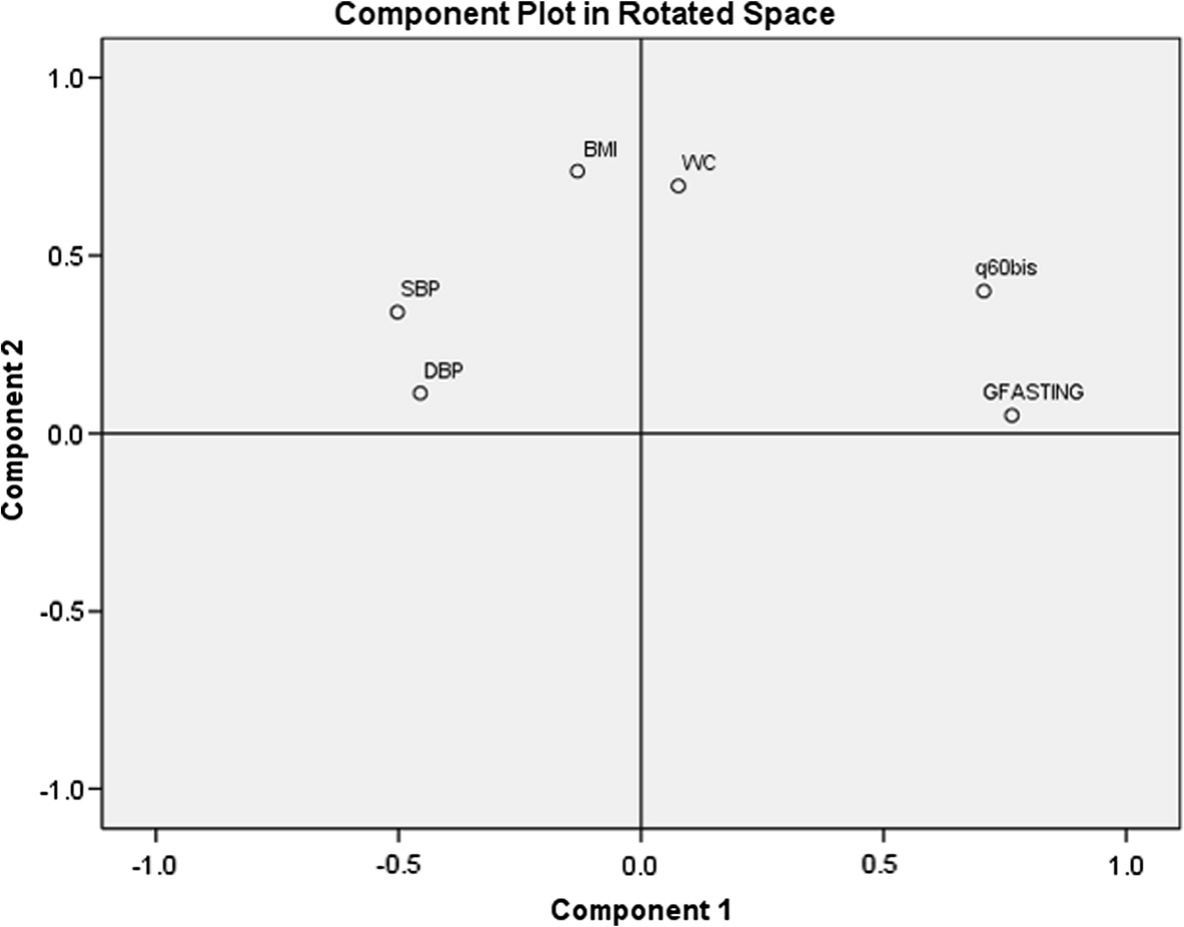
Two-component plot in rotated space among participants without cardiometabolic risk.

Factor analysis revealed two uncorrelated factors that cumulatively explained 48.1% of the observed variance of the absence of CDM. The number of those two factors was determined by the scree plot according to Eigen-value (Figure 1). These two factors could be identified as Blood Glucose Metabolism Disordering (Factor 1; 26.3% of variance) and obesity (Factor 2; 22% of variance) (Table 5 and Figure 2).

**Table 5:** Rotated component matrix in absence of cardiometabolic risk

	**Factor 1**	**Factor 2**
BMI	−0.131	0.738
SBP	−0.502	0.340
DBP	−0.455	0.114
WC	0.077	0.696
FPG	0.765	0.051
Post-Load PG	0.707	0.400

### Presence of CDM

**Figure 3:**
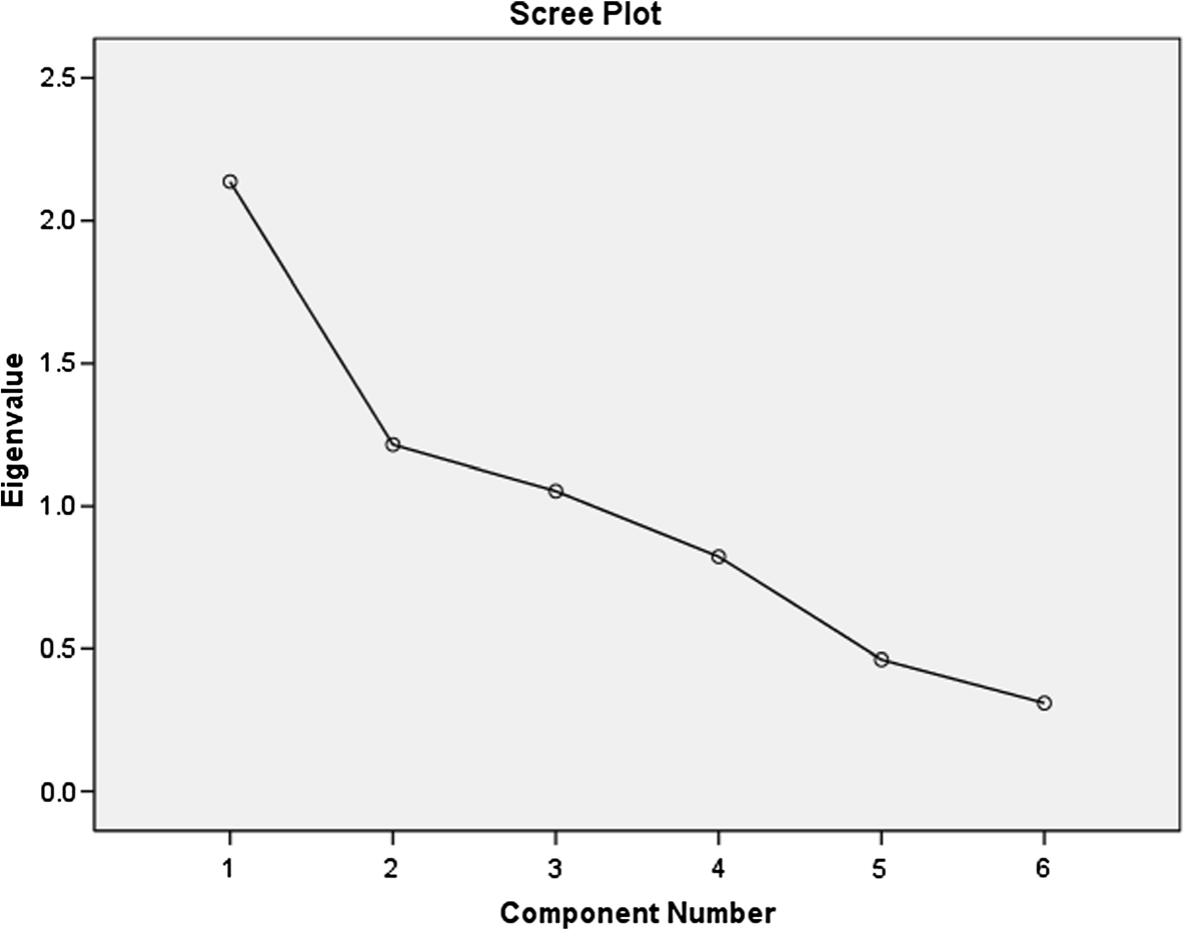
Eigen values among participants with cardio-metabolic risk.

The mean values of variables analyzed in participants with CDM are presented in Table 6. Factor analysis revealed three uncorrelated factors that cumulatively explained 73.6% of the observed variance in the presence of cardiometabolic risk. The number of these three factors (Components) was determined by the scree plot according to Eigen-values (Figure 3).

**Table 6:** In participants with cardiometabolic risk 2

**Variables**	**Mean±SD**
BMI (Kg/m^2^)	43.40 ± 20.20
Post-Load PG (mg/dL)	80.00±15.80
SBP (mmHg)	127.60 ± 26.70
DBP (mmHg)	80.00 ± 15.80
FPG (mg/dL)	93.40 ± 19.80
Waist Circumference (CM)	182.84 ± 101.10

These three factors could be identified as: Blood Glucose Metabolism Disordering (Factor 1; 35.6% of variance), Blood Pressure (Factor 2; 20.3% of variance), and obesity (Factor 3; 17.7% of variance) (Table 7).

**Table 7:** Rotated component matrix in presence of cardiometabolic risk

	**Factor 1**	**Factor 2**	**Factor 3**
BMI	0.077	−0.025	0.760
SBP	−0.064	0.881	−0.137
DBP	−0.224	0.833	0.116
WC	0.025	0.001	0.769
FPG	0.906	−0.106	0.096
Post-Load PG	0.894	−0.179	0.031

## Discussion

The present study identified MS combination for which factor analysis would be appropriate among Bantu Africans. For that reason, the steps involved in performing factor analysis procedure were described. Thus, factor analysis findings using SPSS software have been interpreted.

However, MS is a complex issue in health care. It does not have a simple cause, but multiple risk factors. Its natural course is influenced by genetic factors, personal (Host) attributes, environmental characteristics, or some interactions of both.

At our knowledge, this was the first study to characterize factor analysis of possible risks for clustering of some traditional cardiovascular risk factors in the general population, absence of CDM, presence of CDM, and presence of MS among Bantu Africans living in DR Congo(Central region).

The extent of T2DM, CDM (concurrent presence of 3 non-lipid components of MS), and MS defined by IDF 5 criteria such as 3 non-lipid components and 2 lipid-lipoprotein components [6] was examined. The present study also determined the interrelation of the main CDM factors: BMI, WC, SBP, DBP, FPG, and post-load glucose.

### Emerging burden of MS

Contrary to the previous myths, non communicable diseases (Diabetes, hypertension, MS, atherosclerosis) are no longer rare in Africa [10-14]. The extent is increasing and it is thought to be due to the shifting from traditional African customs to the Western lifestyle [15-18].

#### MS pattern

The present study sought at identifying the physiogenic factors responsible for the clustering of cardiometabolic components. Factor analysis showed marked differences in the MS pattern between the groups of 3 components (CDM) and 5 components (MS).

### Number of generated factors

In the general adult population, factor analysis identified 3 components for MS. This finding about MS was consistent with a study conducted in Asian Indians from the general population [1]. In India, however, the total variance of 65.3% [1] was lower than the total variance of 75.1% explained in the present study. However, in the South African general population, 5 factors could be identified in factor 1(Obesity), factor 2(Hypertension), factor 3(Hyperuricemia-hypertriglyceridemia), factor 4(Hyperglycemia), and factor 5(Hyperinsulinemia) [1]. In our findings, the first 2 factors cumulatively explained 58% of the total variance for MS. Only considering 3 non-lipid components, affordable in limited resources areas, factor analysis had identified also 3 factors with total variance almost 74.5% for CDM and similar with that for MS. The first 2 factors(Dysglycemia and Hypertension) cumulatively explained 56% of the total variance of CDM.

In considering the entire population and the sub-population without CDM, factor analysis generated only 2 factors. In all participants, the factors revealed such as hypertension(factor 1) and dyslipidemia(factor 2) cumulatively explainedb55.4%bof the total variance of the clustering pattern of atherogenic factors from MS. However, in the absence of CDM, BP was not loaded, while only dysglycemia(factor 1) and obesity/BMI and WC(factor 2) were revealed the first factors which cumulatively explained 48.1% of the total variance of the characterization of this group by the clustering of non-lipid components for MS.

The present study showed that no overlapping of variables on more than 1 factor indicated that more than 1 variable was responsible for the ultimate phenotype of the MS. Our findings demonstrated that factor analysis confirmed the general results from other factor analyses of the MS on different ethnic groups that had 3-5 factors revealed [1-4].

Our findings with the clustering of the variables in MS as a result of multiple factors known modifiable in nature raised the following question: would it be more efficient to include all participants in one major factor analysis model? Indeed, factor analysis is practically limited to develop a single-parameter screening tool for MS in this study as mentioned in the literature [4]. IDF recommended WC as the most frequently used anthropometric index to define abdominal obesity [6]. Paradoxically, WC, BP, lipid, and glucose levels were similar among men and women in this study as reported in the same general population [10]. However, WC cutoff points differ by ethnic groups and gender worldwide [1,4]. Older age was associated with T2DM in this study, while age not considered as a component of MS, is a confounding factor for anthropometric variables of MS amon Taiwanese individuals [4].

Factor analysis was applied to see whether there was a less complex space with fewer than the “n” dimensions of the variables that had been analyzed. It was found that a three dimensional space or a mixture of three factors could be used to explain a major part of the data. In more precise mathematical terms the global and examined variables without dyslipidemia(with paradoxes of triglycerides and HDL-C) could be reduced to three factors with eigenvalues greater than one, which explained 73.4% of the variance in MS Africans. The loadings on these factors sorted out into three metabolic groupings.

Neither of the variables was loaded on all the three components. These three factors could be identified as Glucose Metabolism (Factor 1), Blood Pressure (Factor 2) and Obesity (Factor 3). This suggests that those non-lipid components clustered naturally rather than as a result of chance.

No overlapping of variables on more than one factor indicated that more than 1 variable is responsible for the ultimate phenotype of the fats. The present factor analysis confirmed global results from other factor analyses of fats among different populations that had 3 to 4 factors identified as non-modifiable/genetic risk factors and modifiable/ environmental risk factors. The study attempted to observe among BMI, WC, SBP, DBP, FPG, and post-load PG group - which ones go together and which ones do not [30]. Variables with a factor loading of at least 0.3 have generally been considered for interpretation although it is suggested that only loadings ≥ 0.4 be used, which therefore shares at least 15% of the variance with a factor, should be used in the study [24].

In many studies, fats play a pivotal role in the occurrence of the onset of CVD, andT2DM. However, lipid profile and fasting insulin are not available in the majority of health centers in developing countries.

Therefore, identification of non-lipid components of the metabolic syndrome would be helpful in understanding the etiology among Bantu Africans. Virtually no study has been performed on combination of the evaluated variables in Sub- Saharan Africa.

#### Perspectives for Africa

This study highlighted the absence of obesity as a factor of MS in type 2 diabetic Bantu Africans. Moreover, obesity was the third factor of MS with lower variance in comparisons with variances of factor 1(Glucose) and factor 2(Blood pressure) among type 2 diabetic Africans with MS. As reported on the factor analysis of risk variables associated with MS in adult Asian Indians [1], further studies among larger sizes from Bantu Africans, are needed to demonstrate the responsibility of more than one underlying physiogenetic polymorphisms in the present specific glucose-centered pattern for MS with lower BMI and smaller WC.

#### Limitations and strengths

The advantages and disadvantages of factor analysis have been reported in medical, physical, marketing economic and environmental researches [31]. There are different reasons of the limitations of this study, that is, ethnic and cultural heterogeinity, genetic studies, gender, age composition, number of risk variables included, sample size, and cutoff points of MS and CMD[ ]. In Asian Indians, angiotensin converting enzyme gene polymorphism(insertion/deletion) with BP was identified factor 3 along lipids and lipoproteins(factor 1) and centripetal fat and BP(factor 3) associated with MS phenotype [1]. In these Asian Indians, DBP in factor 2 overlapped on another variable in factor 3 [1].

#### Advantages of factor analysis

The rotation methods are useful in making the output more understandable and for ease of interpretation of the factors. The optimal variance of the squared loadings of a factor (Column) on all the variables (rows) in a factor matrix is due to varimax rotation (an orthogonal rotation of the factor axes). Factor matrix differentiates the original variables from extracted factors.

Groups of inter-related variables are identified and seen in their manner to be related to each other.

In multi-factorial diseases, it is easy and inexpensive to perform factor analysis which can be used to identify hidden dimensions which may not be apparent from analysis.

#### Disadvantages of factor analysis

It is not possible to pick the proper rotation using factor analysis alone as all rotations represent different underlying processes and equally valid outcomes of standard factor analysis optimization.

Though not a strictly mathematical criterion, there is much to be said for limiting the number of factors to those whose dimension of meaning is readily comprehensible. The same limitation is reported about variance explained criteria.

The research is requested to choose the solution which generates the most comprehensive evaluation of data.

The Kraiser criterion is the default in SPSS and most computer programs but is not recommended when used as the sole cut-off criterion for estimating the number of factors.

Certain researchers prefer to keep enough factors to account for 80%-90% of the variation. However, other researchers explain variance with a few factors, but lower than 50% (Parsimony).

Factor analysis cannot identify causality as interpreting factor analysis is based on using a “ heuristic” convenient solution even if not absolutely “true”. If important attributes (such as lipid components of fats) at primary health care in developing countries like DRC, the value of the procedure was reduced for BMI in absence of MS.

It requires strong background knowledge of biology and Pathophysiology or theory as multiple attributes may be highly correlated for no apparent reason. Varimax was an orthogonal rotation of the components to maximize the variance of the squared loadings (unrotated output accounted for by the first and subsequent factors) of a dimension (Column) on all the variables(Rows) in a factor matrix. Varimax rotation is the easiest and the most simple and common rotation option used in MS [1-5]. However, oblique rotations might be more suited and more preferred with methods inclusive [31]. In search of underlying dimensions, the use (sometimes an abuse) of factor analysis in Personnality and Social Psychology literature [32]. There are also different rotation methods such as quartimax rotation(an orthogonal alternative), equimax rotation( a compromise between varimax and quartimax criteria), direct oblimin rotation(standard method with a non-orthogonal/oblique rotation with higher eigenvalues but lower interpretability of the factors), and Promax rotation. In this study, we evaluated Promax rotation in addition to varimax rotation. Indeed, Promax rotation was computationally faster alternative non-orthogonal/oblique rotation method than other oblique methods such as direct oblimin rotation. The potential limitations such as the inability of the investigators in collecting sufficient set of product activities, unknown on reasons of associated dissimilar attributes, and obscured factors were excluded or minimized.

#### Implementation of factor analysis

The implementation of Factor analysis is well established within robust statistical software such as SAS, BMDP and SPSS and R programming language with the factanal function (GPA rotations), and Open Opt [33]. This is evidenced by both analysis and scree plots and the three dimensional charts.

## Conclusion

The factor analysis performed for this study suggests that the clustering of the non-lipid variables is sufficient to define CDM in black Africans at including glucose metabolism, Blood pressure and Obesity. Since 3 factors in sequencing dyslipidemia, hypertension, and abdominal obesity-dysglycemia were identified for the Bantu Central African MS phenotype, more one major factor could be accounted for this specific MS. Early prevention and management (diagnosis and proper intervention) strategies for those modifiable loaded risk variables could reduce the burden of type 2 DM, MS, and emerging cardiovascular disease in Central Africa.

## Competing interests

All authors declare that they have no competing interests.

## Authors’ contributions

All the co-authors have seen and approved the final version of the manuscript and it is not currently under active consideration for publication elsewhere, has not been accepted for publication, nor has it been personally and actively involved in substantive work leading to the report, and will hold themselves jointly and individually responsible for its content. JBKLO was responsible for the field work. JNS performed review literature related to factor analysis. BLM conceived of the study, and participated in the study design. JNS and BLM performed statistical analyses. JTL, JNS, BLM, JBKLO, and GT participated in the coordination of writing of the study. All authors read and approved the final manuscript.
